# Chronic Rhinosinusitis Associated with Erectile Dysfunction: A Population-Based Study

**DOI:** 10.1038/srep32195

**Published:** 2016-08-31

**Authors:** Shu-Yu Tai, Ling-Feng Wang, Chih-Feng Tai, Yu-Ting Huang, Chen-Yu Chien

**Affiliations:** 1 Department of Family Medicine, Kaohsiung Medical University Hospital, Kaohsiung Medical University, Kaohsiung, Taiwan; 2 Department of Family Medicine, School of Medicine, College of Medicine, Kaohsiung Medical University, Kaohsiung, Taiwan; 3 Department of Family Medicine, Kaohsiung Municipal Ta-Tung Hospital, Kaohsiung, Taiwan; 4 Research Center for Environmental Medicine, Kaohsiung Medical University, Kaohsiung, Taiwan; 5 Department of Otorhinolaryngology, Kaohsiung Medical University Hospital, Kaohsiung Medical University, Kaohsiung, Taiwan; 6 Department of Otorhinolaryngology, School of Medicine, College of Medicine, Kaohsiung Medical University, Kaohsiung, Taiwan; 7 Department of Otorhinolaryngology, Kaohsiung Municipal Ta-Tung Hospital, Kaohsiung, Taiwan; 8 Department of Otorhinolaryngology, Kaohsiung Municipal Hsiao-Kang Hospital, Kaohsiung, Taiwan; 9 Division of Medical Statistics and Bioinformatics, Department of Medical Research, Kaohsiung Medical University Hospital, Kaohsiung Medical University, Kaohsiung, Taiwan

## Abstract

Few studies have investigated the relationship between chronic rhinosinusitis (CRS) and erectile dysfunction (ED). This case-control study aimed to investigate the association between CRS and the risk of ED in a large national sample. Tapping Taiwan’s National Health Insurance Research Database, we identified people 30 years or older with a new primary diagnosis of CRS between 1996 and 2007. The cases were compared with sex- and age-matched controls. We identified 14 039 cases and recruited 140 387 matched controls. Both groups were followed up in the same database until the end of 2007 for instances of ED. Of those with CRS, 294 (2.1%) developed ED during a mean (SD) follow-up of 3.20 (2.33) years, while 1 661 (1.2%) of the matched controls developed ED, mean follow up 2.97 (2.39) years. Cox regression analyses were performed adjusting for sex, age, insurance premium, residence, hypertension, hyperlipidemia, diabetes, obesity, coronary heart disease, chronic kidney disease, chronic obstructive pulmonary disease, asthma, allergic rhinitis, arrhythmia, ischemic stroke, intracerebral hemorrhage, and medications. CRS was revealed to be an independent predictor of ED in the fully adjusted model (HR = 1.51; 95% CI = 1.33–1.73; *P* < 0.0001).

Chronic rhinosinusitis (CRS) is an inflammatory disease of the upper airway that can impact quality of life. It affects more than 30 million people in the United States, where the annual prevalence of CRS is reported to range from 13% to 16%^1^. The costs associated with treatment of CRS there are estimated to reach about US$4.3 billion annually^1^. Previous studies have suggested that upper airway diseases such as allergic rhinitis (AR) and lower airway diseases such as asthma and chronic obstructive pulmonary disease (COPD) may be associated with an increased risk for erectile dysfunction (ED)^2^,^3^,^4^,^5^. Low-grade subclinical inflammation affects endothelial function and is involved in all stages of the atherosclerotic process^6^. In fact, several studies have associated increased expressions of various markers of inflammation with the onset and severity of ED^7^.

Erectile dysfunction (ED), defined by the National Institutes of Health as “the inability to attain and maintain an erection of sufficient quality to permit satisfactory sexual intercourse,” affects ~5% of the male population in the United States^8^,^9^. More than ten million men there are affected by erectile dysfunction, and worldwide it is estimated to affect 100 million men^8^,^10^.

Some reports have suggested that there is a possible relationship between CRS and ED^11^,^12^. Patients with CRS have been found to be at increased risk of stroke and acute myocardial infarction (AMI)^13^,^14^,^15^. The risk factors for cardiovascular disease and ED are known to be similar^7^,^10^, but there has been no large investigation evaluating the risk of ED after a CRS diagnosis. This study tapped a nationwide population-based database in Taiwan to test the hypothesis that CRS is a risk factor for ED.

## Materials and Methods

### Sample

A retrospective cohort study was conducted using data collected from Taiwan’s National Health Insurance Research Database (NHIRD), an insurance claims database managed by Taiwan’s National Health Research Institute (NHRI) for research purposes. It contains demographic information, diagnoses, health services provided, and healthcare costs and prescription data for almost all outpatients and inpatients as well as patients receiving dental services in Taiwan. Implemented in 1995, the National Health Insurance (NHI) program provides compulsory universal health insurance, covering the delivery of virtually all health care (98%) to the entire population. In cooperation with the Bureau of the NHI, Taiwan’s NHRI extracted a randomly sampled set of representative data for one million people from the 2005 registry of all NHI enrollees to create a subset of the NHIRD for research purposes. The enrollees in this subset, known as the Longitudinal Health Insurance Database (LHID), are not significantly different from all NHI enrollees with regard to age, sex, or health care costs^16^.

CRS was identified in this study by the claims records showing a primary diagnosis of CRS designated with the use of the International Classification of Diseases, Ninth Revision (ICD-9) codes CRS^15^,^17^ (ICD-9 codes: 473, 473.0, 473.1, 473.2, 473.3, 473.8, and 473.9) from 1996 to 2007. Date of diagnosis was set as the index date. Nasal polyps (NP) were identified by a diagnosis of NP ICD-9 codes: 471, 471.0, 471.1, 471.8, and 471.9. CRS without NP (CRSsNP) was defined in subjects who had CRS but no NP. CRS with NP (CRSwNP) was defined in subjects who had CRS with comorbid NP.

The comparison cohort consisted of individuals who had no diagnosis of CRS in the LHID. These individuals were randomly frequency matched with the cases by age and sex at a ratio of 1:10. Their index dates were randomly assigned to months and days within the same year of the index date of the patients in the CRS cohort. We excluded subjects with pre-existing ED (ICD-9 code: 607.84) in both groups.

Both CRS cases and controls were followed up until they were diagnosed with ED, our outcome. ED cases were identified based on a recorded diagnosis of ED ICD-9 code 607.84. All medical claims containing this diagnostic code between 1996 and 2007 were collected from the NHIRD for further analysis. To improve the diagnostic validity of ED, only those patients who had been diagnosed with ED at least twice in an outpatient service or once in an inpatient service by urologists were included in the analysis.

Physicians in Taiwan generally adhere to standardized CRS definitions and management guidelines, such as those provided by the Rhinosinusitis Task Force^18^ and Clinical Practice Guidelines: Adult Sinusitis^19^. The diagnosis of ED in Taiwan is based on criteria established by the American Urological Association^20^,^21^.

The covariates considered in this analysis were sex, age, insurance premium, area of residence (metropolitan, class I cities, class II cities, class I counties, class II counties, and unclassified), hypertension, hyperlipidemia, diabetes, obesity, coronary heart disease, chronic kidney disease (CKD), chronic obstructive pulmonary disease (COPD), asthma, allergic rhinitis, arrhythmia, ischemic stroke, intracerebral hemorrhage, and medications [angiotensin-converting enzyme inhibitors (ACEIs), beta-adrenergic blockers, statins, and steroid]. The insurance premium served as an indicator of economic status and subjects were classified into 1 of 3 categories: fixed premium and dependent, NTD20,000 (income per month) or less, and more than NTD20,000 (US$1 = NTD32.8 in 2007).

This study was exempt from full review by the Institutional Review Board of Kaohsiung Medical University Hospital because the NHIRD only contains de-identified secondary data released to the public for research purposes (KMUHIRB-EXEMPT-20150003).

### Statistical analysis

The baseline characteristics and comorbidities of the CRS cohort and non-CRS cohort were analyzed descriptively. All data were expressed as mean ± standard deviation (SD) or percentage. Chi-square and *t*-tests were used to test differences in categorical and continuous variables between the two cohorts. Univariate and multivariable Cox proportional hazards regression were used to assess the hazard ratio (HR) and 95% confidence interval (CI) of ED associated with CRS, compared with the non-CRS cohort. The multivariable analysis model was adjusted for sex, age, insurance premium, residence, hypertension, hyperlipidemia, diabetes, obesity, coronary heart disease, chronic kidney disease (CKD), chronic obstructive pulmonary disease (COPD), asthma, allergic rhinitis, arrhythmia, ischemic stroke, intracerebral hemorrhage, and medications of ACEIs, beta-adrenergic blockers, statins, and steroid. This study also investigated the relationship between CRS w/s NP and ED as well as CRS and ED. To find out if CRS is an age-dependent risk factor for ED, we also analyzed the effect of CRS on ED stratified by age group.

The cumulative incidence of ED between the CRS cohort and the non-CRS cohort was analyzed using the Kaplan–Meier method, and the difference was examined by log-rank test. SAS software (version 9.3 for Windows; SAS Institute Inc., Cary, NC) was used for all statistical operations. A *P* value < 0.05 was considered significant.

## Results

### Characteristics of the subjects

The two cohorts were comprised of 14 039 people with newly diagnosed CRS and 140 387 (nearly ten for every patient in the CRS group) sex-, age-matched controls, both identified from the NHIRD 1996–2007. As can be seen in Table 1, a summary of cohort characteristics, most cases of CRS occurred in people 36–50 years old. Compared to the controls, more patients with CRS were diagnosed with hypertension, hyperlipidemia, diabetes, obesity, coronary heart disease, chronic kidney disease (CKD), chronic obstructive pulmonary disease (COPD), asthma, allergic rhinitis, arrhythmia, ischemic stroke, and intracerebral hemorrhage (*P* < 0.0001) (Table 1). The patients in the CRS cohort were also more frequent users of ACEIs, beta-adrenergic blockers, statins, and steroid during the follow-up period than those without (*P* < 0.0001) (Table 1).

### Association between CRS and risk of ED

Of the 154 426 subjects, 1955 (cases 294, 2.1%; controls 1661, 1.2%) were diagnosed with ED during the follow-up period. Twenty patients (2.6%) in the CRSwNP cohort and 274 (2.1%) in the CRSsNP cohort were later diagnosed with ED. The mean (SD) follow-up interval for the CRS cohort was 3.20 (2.33) years and for the non-CRS cohort was 2.97 (2.39) years. As shown in Table 2, CRS was positively associated with ED (HR = 1.51; 95% CI = 1.33–1.73; *P* < 0.0001) in the fully adjusted Cox regression model adjusted for sex, age, insurance premium, residence, hypertension, hyperlipidemia, diabetes, obesity, coronary heart disease, chronic kidney disease (CKD), chronic obstructive pulmonary disease (COPD), asthma, allergic rhinitis, arrhythmia, ischemic stroke, intracerebral hemorrhage, and medications.

### CRS w/s NP and risk of ED

Both CRSwNP and CRSsNP were associated with ED (HR = 1.78; 95% CI = 1.15–2.78; *P* = 0.0106 and HR = 1.50; 95% CI = 1.31–1.72, respectively; *P* < 0.0001) in the same fully adjusted Cox regression models. In the stratified analysis, however, there was no significant difference between the associations of CRSsNP and CRSwNP with ED [contrast of CRSsNP versus CRSwNP, estimate = 0.84 (0.54–1.32), *P*-value = 0.4552] (Table 2).

### Stratified by age

To evaluate whether CRS is an age-dependent risk factor for ED, we divided the CRS patients into four age groups: 30–35 years, 36–50 years, 51–65 years, and >65 years. After adjusting Cox regression models for potential confounding factors, the highest adjusted HR for ED in the patients with CRS compared with the controls was 1.71 (95% CI = 1.36–2.14, P < 0.0001) in the patients aged 36–50 years (Table 3).

**Figure 1:**
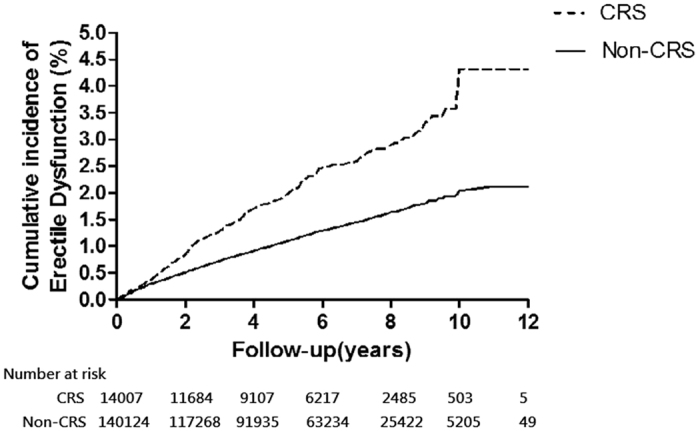
Cumulative incidence on erectile dysfunction by study groups [CRS (dashed line) VS Non-CRS (solid line)]. Modified log-rank test *p* value < 0.0001.

**Figure 2:**
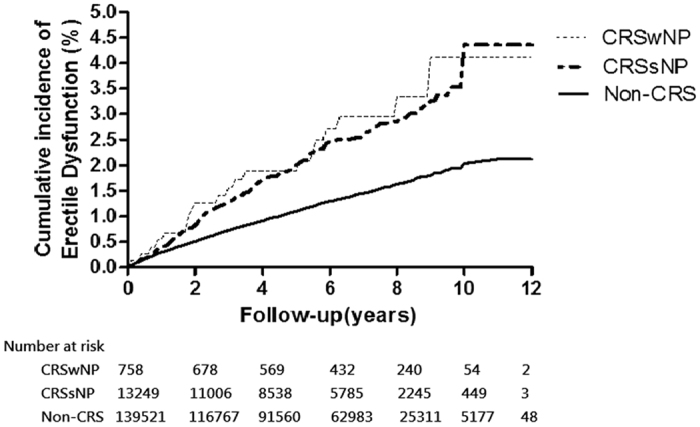
Cumulative incidence of erectile dysfunction in CRSwNP (fine dashed line), CRSsNP (thick dashed line), and Non-CRS (solid line). Modifed log-rank test *p* value < 0.0001.

Kaplan–Meir analysis of cumulative incidence from ED revealed that patients with CRS had a significantly higher incidence of ED than the non-CRS group (*P* < 0.0001, based on a modified log-rank test) (Fig. 1). The cumulative incidence of ED in the CRS w/s NP cohort was also higher than that in the non-CRS cohort (*P* < 0.0001) (Fig. 2).

## Discussion

In this large-scale nationwide study using claims data from Taiwan, we observed that although comorbid diseases were more likely to be prevalent in the CRS cohort than in the control cohort, CRS remained an independent risk factor for the development of ED after adjustment for covariates. Patients with CRS were found to be at significantly increased risk of ED (HR = 1.51; 95% CI = 1.33–1.73; *P* < 0.0001), after adjustment for possible confounding factors.

Treatment of CRS has been found to improve ED. A hospital-based study by Gunhan *et al*. showed that ED was more common in patients with CRSwNP than in control patients, subjectively measured using the International Index of Erectile Function (IIEF-EF) (*P* = 0.009) and objectively measured by nocturnal penile tumescence (NPT) (*P* = 0.0118)^12^. Their results indicated the presence of ED in 34% (IIEF-EF) and 24% (NPT) of their patients with CRSwNP. After comparing the pre- and postoperative results of the CRSwNP patients, the authors further found erectile function to be significantly improved after functional endoscopic sinus surgery^12^. A hospital-based study by Benninger *et al*., who used the Rhinosinusitis Disability Index, a validated instrument that measures the physical, functional, and emotional impact of CRS on a person’s quality of life (QoL), found that patients with CRS had significantly improved sexual function scores after surgery for their CRS (*P* < 0.001)^11^.

The exact mechanism underlying the development of ED in CRS patients remains unknown. The relationship between CRS and ED could conceivably be influenced by a number of pathophysiologic factors, including QoL, hypoxia, and inflammation. The inflammatory biomarker and cytokines that link CRS and ED may include C-reactive protein (CRP), tumor necrosis factor alpha (TNF-α), and transforming growth factor beta (TGF-β). Some consequences of CRS, including low QoL, hypoxia, and cardiovascular disorders, might increase the risk of ED in a select subpopulation of patients with CRS.

Videler *et al*., administering the Short-Form health survey (SF-36), found that patients with CRS had significantly worse QoL scores than patients with other chronic conditions, including head and neck cancer, hypertension, angina pectoris, and migraine^22^. Interestingly, Ottaviano *et al*. observed a significant association between olfactory sensitivity and sexual desire in young adults (*P* = 0.02)^23^. Thus, the CRS symptoms of nasal obstruction, rhinorrhea, smell dysfunction, facial pressure, headache, and postnasal drainage might reduce libido leading to ED.

Ozdemir *et al*. found that patients with CRSwNP had hypoxia and nasal surgery led to increased blood oxygen saturation^24^. Hypoxia increases afferent sympathetic activation, resulting in increased vasoconstriction and significantly reduced nitric oxide (NO) synthase activity, which is a rate-limiting factor for NO production in the penile corpus cavernosum^25^. Studies have suggested that NO is a bronchodilator and vasodilator involved in mucocililary-regulating activities^26^. Lindberg *et al*. reported that their patients with CRS had lower concentrations of nasal NO than their healthy control patients^27^. Studies have also shown an inverse correlation between nasal NO and nasal polyp size^28^. Ragab *et al*. observed that nasal NO levels increased after the medical and surgical treatment of CRS^26^. NO plays a crucial role in the molecular mechanism of penile smooth muscle relaxation^9^, and decreased levels of NO can contribute to ED. In addition, Verratti *et al*. revealed that men who are sexually potent at sea level had pathological rigidometric records at a high altitude in a hypoxic environment^25^. Therefore, a selected subpopulation of CRS patients with hypoxia may develop ED.

Recent epidemiological studies have identified an increased risk of subsequent stroke and acute myocardial infarction in patients with CRS^13^,^14^,^15^. In previous studies on CRS patients, the HRs of stroke, ischemic stroke, and acute myocardial infarction were 1.34 (95% CI = 1.04–1.74)^13^, 1.34 (95% CI = 1.18–1.53), and 1.48 (95% CI = 1.32–1.67)^15^, respectively, after adjustment for potential confounding factors. CRS is defined as a group of disorders associated with inflammation of the nasal mucosa and sinuses lasting at least 12 weeks^29^. Inflammation is thought to play a central role in the pathogenesis of atherosclerotic initiation, plaque rupture, thrombosis, and stroke^30^. In the first stages of atherosclerosis, the penis is typically first affected, manifesting as ED, making ED a possible warning sign that a heart attack or stroke is imminent, often within 3 to 5 years^31^. Thompson *et al*. proposed that ED is a sentinel symptom in patients with occult cardiovascular disease^10^, suggesting that CRS could be a risk factor for ED.

Elevation of the systemic inflammatory marker, CRP, has been reported to correlate significantly with increasing severity of penile vascular disease in men with ED as measured by penile Doppler^32^. In a study by Giugliano *et al*., circulating CRP levels were significantly higher in obese men with ED than in obese men without ED (*P* < 0.05)^33^. In other studies, patients with sinusitis have had higher CRP levels compared with control patients^34^,^35^. Hirshoren *et al*. observed a significant association between CRP levels and the severity of sinusitis^36^, suggesting that CRP might play a direct role in the pathogenesis of atherosclerosis. These findings have led to the hypothesis that CRS might induce local inflammation in the upper airway and induce systemic inflammatory effects that contribute to ED. CRP might thus play a role in both CRS and ED.

TNF-α might also play a key role in inducing ED or contribute to the pathophysiology of ED. Oyer *et al*. reported that the mucus of patients with CRSwNP had higher levels of the proinflammatory cytokine TNF-α than their control patients^37^. Carneiro *et al*. found increased serum TNF-α levels in patients with moderate to severe ED^38^.

The fibrogenic cytokine TGF-β plays key roles in CRS and ED. Van Crombruggen *et al*. observed upregulated TGF-β expression in patients with CRSsNP^39^. In a study by Ryu *et al*., plasma TGF-β expression was significantly higher in ED patients than in control patients^40^. Previous studies have also revealed that TGF-β is involved in cavernous fibrosis, promoting the development of vasculogenic ED in men^41^.

Our results indicate a stronger association between CRS and ED men aged ≥35 years than in other age groups, particularly in men aged 36–50 years (Table 3). However, the mechanism underlying these age-associated effects remains unclear.

### Strengths and limitations

The key strengths of this study are its nationally representative sample, physician-based diagnosis to identify CRS cases, longitudinal analysis, and range of covariates considered, including sociodemographic factors and general health status. However, this study has some limitations, particularly those caused by the use of routine claims data, which do not include information on other potential confounders such as severity of CRS status, stressful life events, smoking, air pollution, body mass index, and family history. This study was conducted in Taiwan. Whether our findings can be extrapolated to other countries is not known.

## Conclusion

In summary, this study found an association between having CRS and a significantly higher risk of developing ED than the general population, regardless of age, presence of comorbidity, and medications. Because of the high (and potentially increasing) prevalence of CRS, clinicians should be aware of the risk of ED in CRS patients. Further research is required to determine the underlying mechanism linking these two adverse health outcomes.

## Additional Information

**How to cite this article**: Tai, S.-Y. *et al*. Chronic Rhinosinusitis Associated with Erectile Dysfunction: A Population-Based Study. *Sci. Rep.* **6**, 32195; doi: 10.1038/srep32195 (2016).

## Figures and Tables

**Table 1:** Characteristics of CRS cases and matched^a^ controls in Taiwan, 1996–2007.

Characteristic	Class	Total	CRS		Non-CRS^a^		P-value
			N	%	N	%	
Total		154426	14039	9.1	140387	90.9	
Age group	30–35	27684	2518	17.9	25176	17.9	1.0000
	36–50	68050	6185	44.1	61865	44.1	
	51–65	34418	3130	22.3	31288	22.3	
	>65	24264	2206	15.7	22058	15.7	
Follow-up time (years) (Mean ± SD)			3.20 ± 2.33		2.97 ± 2.39		0.1396
Insurance premium	Fixed premium and dependent	20386	1722	12.3	18664	13.3	<0.0001
	≤NTD^b^ 20,000	78974	6453	46.0	72521	51.7	
	>NTD20,000	55066	5864	41.8	49202	35.0	
Residence	metropolitan	39079	3633	25.9	35446	25.2	<0.0001
	class I cities	12480	1289	9.2	11191	8.0	
	class II cities	6519	725	5.2	5794	4.1	
	class I counties	67369	6324	45.0	61045	43.5	
	class II counties	27830	1982	14.1	25848	18.4	
	Unclassified	1149	86	0.6	1063	0.8	
Comorbidities							
Hypertension^c^	No	132635	11383	81.1	121252	86.4	<0.0001
	Yes	21791	2656	18.9	19135	13.6	
Hyperlipidemia^d^	No	144244	12687	90.4	131557	93.7	<0.0001
	Yes	10182	1352	9.6	8830	6.3	
Diabetes mellitus^e^	No	144497	12985	92.5	131512	93.7	<0.0001
	Yes	9929	1054	7.5	8875	6.3	
Obesity^f^	No	154234	14002	99.7	140232	99.9	<0.0001
	Yes	192	37	0.3	155	0.1	
Coronary heart disease^g^	No	146359	12920	92.0	133439	95.1	<0.0001
	Yes	8067	1119	8.0	6948	4.9	
Chronic kidney disease (CKD)^h^	No	151382	13616	97.0	137766	98.1	<0.0001
	Yes	3044	423	3.0	2621	1.9	
Chronic obstructive pulmonary disease (COPD)^i^	No	145941	12000	85.5	133941	95.4	<0.0001
	Yes	8485	2039	14.5	6446	4.6	
Asthma^j^	No	150035	12939	92.2	137096	97.7	<0.0001
	Yes	4391	1100	7.8	3291	2.3	
Allergic rhinitis^11^	No	145077	10223	72.8	134854	96.1	<0.0001
	Yes	9349	3816	27.2	5533	3.9	
Arrhythmia^l^	No	149443	13188	93.9	136255	97.1	<0.0001
	Yes	4983	851	6.1	4132	2.9	
Ischemic stroke^m^	No	151753	13729	97.8	138024	98.3	<0.0001
	Yes	2673	310	2.2	2363	1.7	
Intracerbral hemorrhage^n^	No	150882	13594	96.8	137288	97.8	<0.0001
	Yes	3544	445	3.2	3099	2.2	
Medications							
ACEIs	No	148450	13348	95.1	135102	96.2	<0.0001
	Yes	5976	691	4.9	5285	3.8	
Beta-adrenergic blockers	No	146248	12932	92.1	133316	95.0	<0.0001
	Yes	8178	1107	7.9	7071	5.0	
Statins	No	151899	13727	97.8	138172	98.4	<0.0001
	Yes	2527	312	2.2	2215	1.6	
Steroid	No	119028	8629	61.5	110399	78.6	<0.0001
	Yes	35398	5410	38.5	29988	21.4	
Development of ED^o^ during follow-up		1955	294	2.1	1661	1.2	<0.0001

^a^Matched by sex and age (±1 years old).

^b^1US$ = 32.8 NTD in 2007.

^c^ICD-9: Hypertension (401-405).

^d^ICD-9: Hyperlipidemia (272.2, 272.4).

^e^ICD-9: Diabetes mellitus (250).

^f^ICD-9: Obesity (278, 278.0, 278.00, 278.01).

^g^ICD-9: Coronary heart disease (410-414, 429.2).

^h^ICD-9: Chronic kidney disease (CKD) (580-587).

^i^ICD-9: Chronic obstructive pulmonary disease (COPD)(491, 492, 494, 496).

^j^ICD-9: Asthma (493).

^k^ICD-9: Allergic rhinitis (477, 477.0, 477.1, 477.8, 477.9).

^l^ICD-9: Arrhythmia (427, 785.0, 785.1).

^m^ICD-9: Ischemic stroke (433-434, 436, 437.1).

^n^ICD-9: Intracerbral hemorrhage (430-462.9).

^o^ICD-9: Erectile dysfunction (ED) (607.84).

**Table 2:** Adjusted Cox regression analyses on erectile dysfunction (ED) in Taiwan, 1996–2007.

	Crude HR^a^	95% CI^b^	P-value	Adjusted HR	95% CI^b^	P-value
Non-CRS	1	—	—	1	—	—
CRS	1.78	1.57–2.02	<0.0001	1.51	1.33–1.73	<0.0001
CRSsNP	1.77	1.56–2.01	<0.0001	1.50	1.31–1.72	<0.0001
CRSwNP	1.95	1.26–3.04	0.0029	1.78	1.15–2.78	0.0106

^*^Contrast of CRSsNP versus CRSwNP, Estimate = 0.84 (0.54–1.32), *P*-value = 0.4552.

^a^HR: hazard ratio.

^b^CI: Confidence interval.

**Table 3:** Effect^a^ of CRS on ED in Taiwan by age group, 1996–2007.

Development of ED	Age group											
	30–35			36–50			51–65			>65		
	Study group	Comparison	P-Value	Study group	Comparison	P-Value	Study group	Comparison	P-Value	Study group	Comparison	P-Value
	N (%)	N (%)		N (%)	N (%)		N (%)	N (%)		N (%)	N (%)	
Yes	19 (0.76)	121 (0.48)		105 (1.70)	512 (0.83)		94 (3.01)	584 (1.87)		76 (3.45)	444 (2.02)	
Crude HR^b^ (95% CI^c^)	1.57 (0.97–2.55)	1	0.0672	2.07 (1.68–2.55)	1	<0.0001	1.62 (1.31–2.02)	1	<0.0001	1.73 (1.35–2.20)	1	<0.0001
Adjusted HR (95% CI)	1.39 (0.83–2.34)	1	0.2134	1.71 (1.36–2.14)	1	<0.0001	1.27 (1.03–1.60)	1	0.0485	1.47 (1.14–1.91)	1	0.0036

^a^Adjusted Cox regression analyses controlling by sex, age, insurance premium, residence, hypertension, hyperlipidemia, diabetes, obesity, coronary heart disease, chronic kidney disease, chronic obstructive pulmonary disease, asthma, allergic rhinitis, arrhythmia, ischemic stroke, intracerebral hemorrhage, and medications.

^b^HR: hazard ratio.

^c^CI: Confidence interval.
